# Parvovirus-Induced Transient Aplastic Crisis in a Patient With Newly Diagnosed Hereditary Spherocytosis

**DOI:** 10.7759/cureus.8995

**Published:** 2020-07-03

**Authors:** Nitish Singh Nandu, Husam Hafzah, Charmi Patel

**Affiliations:** 1 Internal Medicine, Chicago Medical School, Rosalind Franklin University, North Chicago, USA; 2 Internal Medicine, Rosalind Franklin University of Medicine and Science, North Chicago, USA

**Keywords:** parvovirus, hereditary spherocytosis, aplastic crisis

## Abstract

Parvovirus B19 infections are prevalent in children and commonly present as slapped cheek fever, also known as the fifth disease. They are seen frequently in daycares and professions that require close contact with children. The most common presentation is a rash that is prominent on the cheeks; less common symptoms include painful or swollen joints (polyarthopathy syndrome). The infection is self-limited and resolves within one to two weeks. The virus has an affinity to the red blood cell (RBC) precursors and can rarely cause temporary cessation of the bone marrow's RBC production, leading to aplastic anemia. This is especially of significance in patients predisposed to increased RBC destruction, such as hereditary spherocytosis, sickle cell anemia, and other morphological abnormalities of the RBC. The overlapping arrest of RBC production and excessive destruction leads to a transient aplastic crisis (TAC), leading to severe life-threatening anemia, requiring blood urgent blood transfusions. There have been many studies reporting the incidence of TAC in patients with sickle cell crisis. Only a few cases have been reported in patients with hereditary spherocytosis.

## Introduction

Human parvovirus is a non-enveloped single-stranded DNA virus from the Parvoviridae family and is the only member known to be pathogenic to humans. The virus is transmitted by respiratory droplets and seen commonly in children. Parvovirus b19 infection is prevalent with seropositivity in up to 60% by age 30 years. It causes erythema infectiosum in immunocompetent children but can manifest as pure red cell aplasia in the immunocompromised [1]. Parvovirus directly attacks and destroys proerythroblasts by attaching to the blood group P antigen receptor [2]. Transient aplastic crisis (TAC) is a temporary cessation of red cell production by the bone marrow leading to severe anemia, most commonly seen in patients predisposed to increased RBC destruction. Hereditary spherocytosis is a membrane defect of the RBC, which causes the loss of the standard biconcave shape leading to spherical RBC, spherocytes. These RBCs are easily prone to destruction while passing through the capillaries. When TAC occurs in such patients, the destruction of circulating cells, along with the lack of new cell production, leads to severe anemia that can be life-threatening. There have been many studies reporting the incidence of TAC in patients with sickle cell crisis. Only a handful of cases have been reported in hereditary spherocytosis [2-4]. We report an example of a TAC revealing undiagnosed hereditary spherocytosis in a 23-year-old patient.

## Case presentation

**Figure 1:**
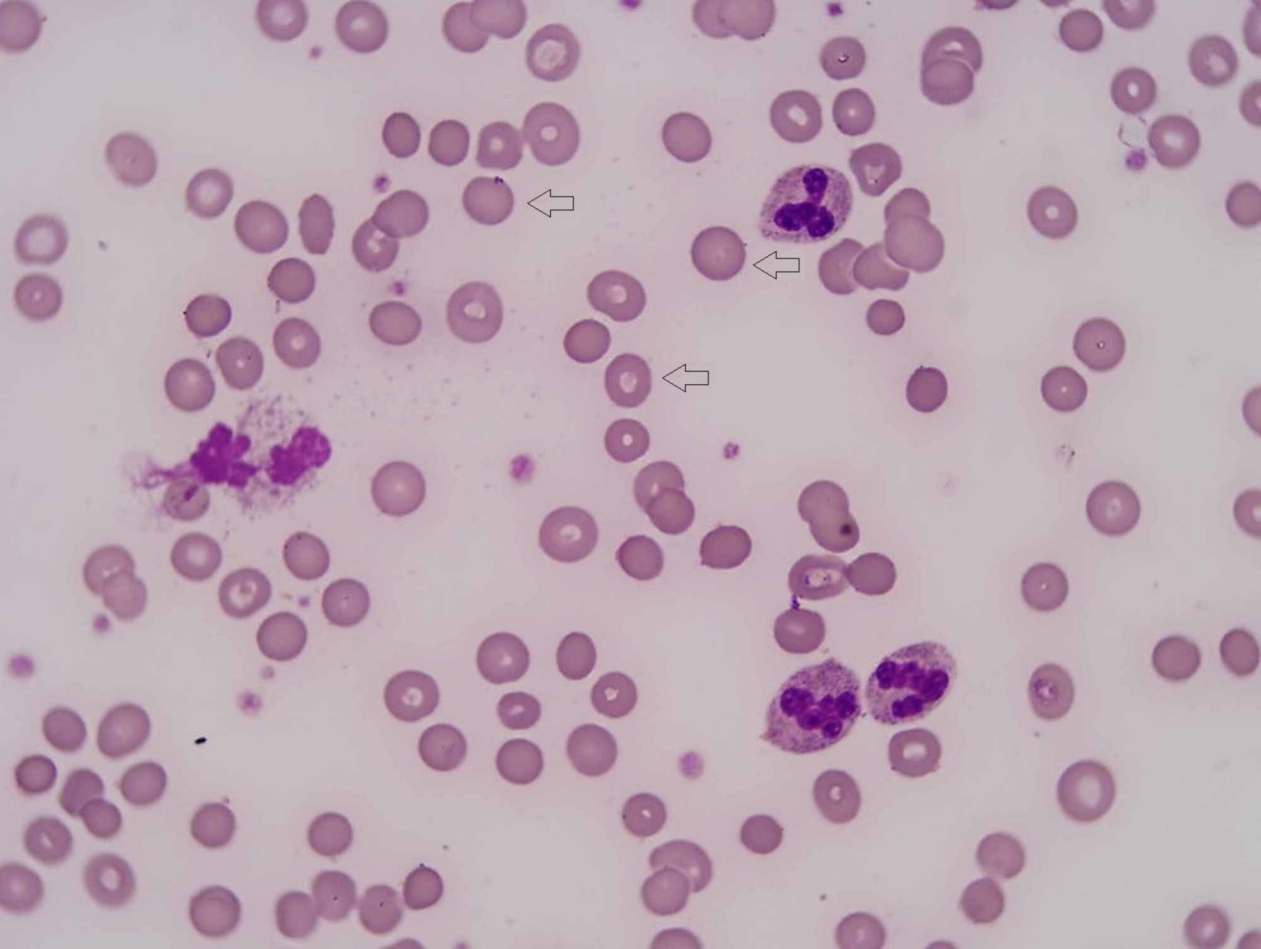
Peripheral blood smear showing spherocytosis (red arrows) Red arrows: spherocytes in the patient's peripheral smear showing dense staining and small rounded appearance

A 23-year-old male was brought to the emergency room (ER) for evaluation after a syncopal episode. The episode was without involuntary shaking, tongue biting, and bowel or bladder incontinence. He had no similar episodes in the past. He reported no significant past medical history. However, he reported cough, sore throat, subjective fever, and chills one week before the presentation. Family history was significant for hereditary spherocytosis in his mother and younger brother. He had a blood pressure of 117/66 mmHg, a heart rate of 98/min, and a temperature of 98.7°F. Upon physical exam, he had a bruise on the left side of his face from the fall, and enlarged tonsils with grayish exudates. There was no rash, lymphadenopathy, or hepatosplenomegaly. Labs showed hemoglobin and hematocrit of 5.0 g/dL and 13.6%, respectively. One week prior, they were 11.8 g/dL and 33.4%. His white blood cell (WBC) count was 8.7 K/µL, and the platelet count was 146 K/µL. His lactate dehydrogenase (LDH) was elevated to 368 U/L, total bilirubin to 2.2 with indirect bilirubin of 2.0, a reticulocyte number of <10 K/µL, and a haptoglobin of <7.75 mg/dL. He had a B12 of 474 pg/mL and serum folate of 13.6 ng/mL. Iron panel showed a low serum iron 284 µg/dL, normal total iron binding capacity (TIBC) 320 µg/dL, high iron saturation 89%, and normal serum ferritin 122.6 ng/mL. The peripheral blood smear showed spherocyte (Figure 1). Chest x-ray showed small left lower lobe pneumonia.

**Figure 2:**
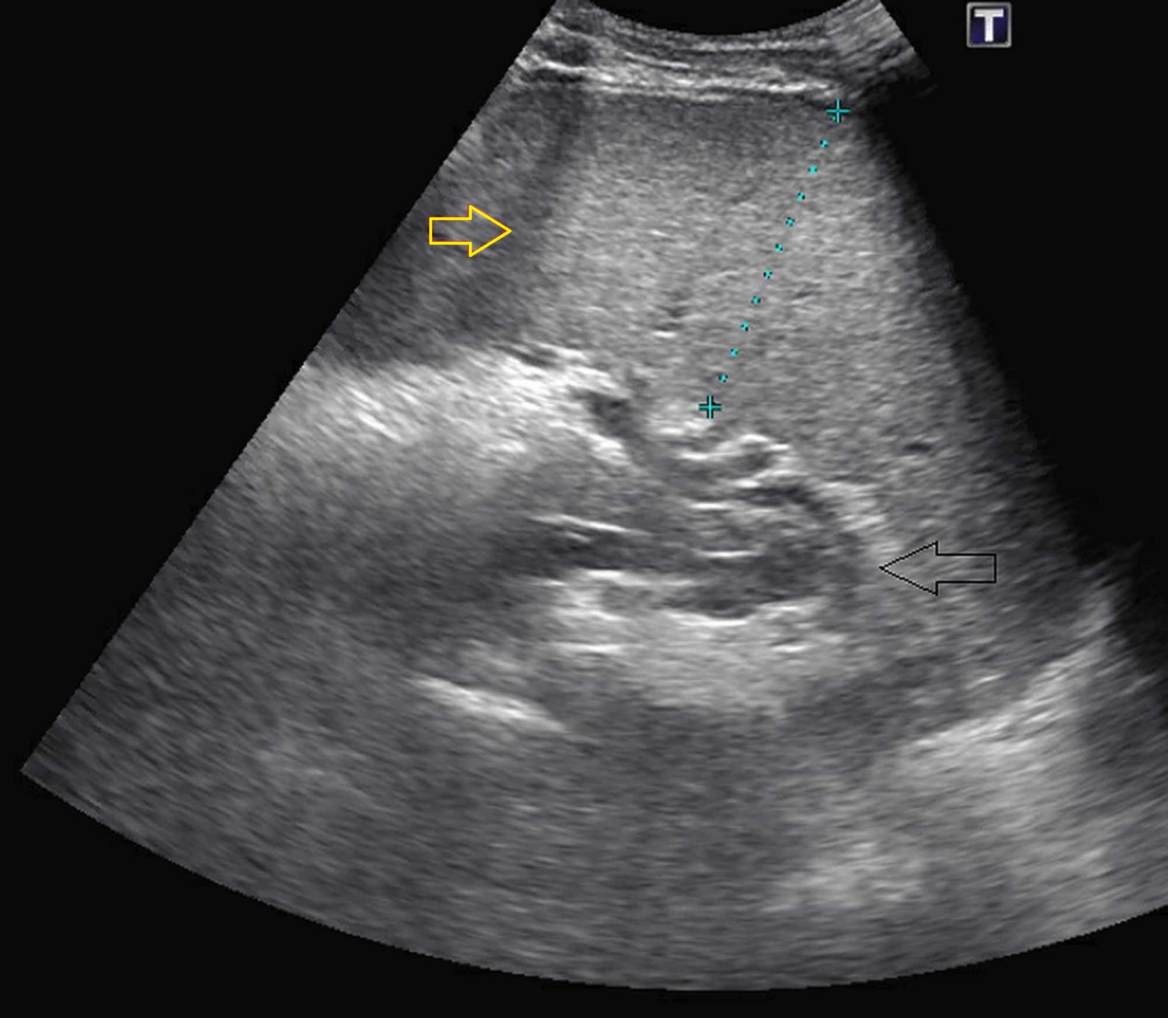
Patient's right upper quadrant ultrasound showing splenomegaly (yellow arrow) Yellow arrow: enlarged spleen; black arrow: stomach

Ultrasound revealed moderate splenomegaly (measuring 17.5 cm x 6 cm x 5 cm), and coarse echotexture of the hepatic parenchyma secondary to fatty infiltration (Figure 2). CT of the head was normal, and a CT of the maxillary sinuses showed mild sinusitis in the maxillary sinuses. The patient was transfused four units of blood and was given IV antibiotics. He was later found to be positive for parvovirus DNA by PCR and Epstein-Barr virus (EBV) IgG and IgM. An osmotic fragility test was positive, confirming the diagnosis of hereditary spherocytosis. He tested negative for cold agglutinin, direct antiglobulin, serum protein electrophoresis, and urine hemosiderin. The patient’s condition improved over time with supportive care and close monitoring. On follow-up, his hemoglobin improved to 10 g/dL, hematocrit to 33.6%, reticulocyte count increased to 261.9 K/µL, LDH 158 U/L, and a haptoglobin <7.75 mg/dL. 

## Discussion

Human parvovirus is a non-enveloped single-stranded DNA virus from the Parvoviridae family and is the only member known to be pathogenic to humans. The virus is transmitted by respiratory droplets and seen commonly in children. It causes erythema infectiosum in immunocompetent children but can manifest as pure red cell aplasia in the immunocompromised [1]. Through the blood group P antigen receptor, it infects and destroys the erythroid progenitor cells inhibiting erythropoiesis, leading to acute erythroblastopenia and reticulocytopenia [2,3]. TAC is generally seen in older individuals, and the most common presentation is fever, lethargy, and non-specific flu-like symptoms. A significant drop in hemoglobin and hematocrit can be observed, particularly in patients with hereditary spherocytosis, sickle cell, and other structural abnormalities of the RBC. The anemia can be drastic, requiring urgent blood transfusions. A bone marrow biopsy would show severe red cell aplasia and giant pronromoblast with viral inclusions. Although TAC affects the erythroid cell lines, WBCs and platelets may also decline [4]. There are only a few documented cases with parvovirus and EBV coinfection, and the latter is most likely responsible for the splenomegaly observed in our patient. The specific role of coinfection on the severity of the aplastic crisis remains unclear [5]. TAC is a relatively self-limited infection that usually resolves in one to two weeks and confers a lasting immunity in immunocompetent individuals [4]. Immunocompromised patients are potentially at risk for reinfection and require treatment with intravenous immunoglobulin (IVIG) [6]. In general, most patients recover with supportive care and timely blood transfusions. 

## Conclusions

TAC is a rare and life-threatening condition associated with parvovirus infection. This condition is particularly worrisome for patients with disorders of RBC morphology, such as hereditary spherocytosis. The presentation can be non-specific, as seen in our patient, with a family history of hereditary spherocytosis, presented after a syncopal episode. He was appropriately managed with supportive care, and timely blood transfusions are required. It is vital to consider TAC in parents and professions that involve child care. If missed, TAC can have dire consequences.
